# Integrated multi-omics and machine learning identify EFNA3 as a key biomarker of tumor invasion

**DOI:** 10.3389/fimmu.2026.1742502

**Published:** 2026-04-02

**Authors:** Guangchun Li, Shihao Shi, Qiong Wu, Zhaosheng Chen, Zhen Zhang

**Affiliations:** 1 Department of Gastroenterology, the Second Qilu Hospital of Shandong University, Jinan, Shandong, China; 2 The Second Clinical College of Shandong University, Jinan, Shandong, China

**Keywords:** gastric cancer, immunotherapy, machine learning, multi-omics, tumor microenvironment

## Abstract

**Introduction:**

Gastric cancer remains a major global health burden, and its pronounced molecular heterogeneity hampers progress in precise subtyping and targeted therapy. Invasion-related programs are considered central drivers of malignant progression, yet cross-scale and pan-cancer evidence remains limited. This study aimed to stratify gastric cancer samples by invasion and, within a multi-omics framework, comprehensively assess their clinical features to identify stable biomarkers with translational potential.

**Methods:**

By integrating multiple machine-learning algorithms with multi-omics data, we identified EFNA3 as a core gene closely associated with the invasive phenotype. We further assessed its distribution in malignant cellular subpopulations, its biological associations, and its relevance across multiple cancer types. In vitro and in vivo experiments were performed to validate its functional roles.

**Results:**

EFNA3 was enriched in specific malignant cellular subpopulations and involved in regulating malignant cell development, accompanied by enhanced cell-adhesion, DNA-metabolic, and cell-cycle programs. EFNA3 was consistently overexpressed across multiple cancer types and associated with tumor progression and poor survival. Its expression also coincided with reduced infiltration of effector immune cells and downregulation of immune checkpoints, indicating an immunosuppressive microenvironment. *In vitro* and *in vivo* experiments further demonstrated that EFNA3 promotes gastric cancer cell proliferation, migration, and tumor growth.

**Discussion:**

Collectively, EFNA3 is closely linked to pro-tumor processes and immune evasion in gastric and multiple other cancers, supporting its value as a subtype biomarker and potential therapeutic target.

## Introduction

Gastric cancer continues to exhibit high incidence and mortality worldwide, and its pronounced molecular heterogeneity limits the ability of conventional staging to capture biological complexity, thereby constraining precision diagnosis and therapy (1–4). Clinical practice and multi-omics studies suggest that invasion and metastasis programs permeate the development of gastric cancer. However, cross-scale and pan-cancer evidence and translatable biomarkers remain insufficient (1, 5, 6). Invasion-related pathways (e.g., cell-adhesion remodeling, cell cycle and DNA metabolism, stress and metabolic adaptation) are considered major drivers of malignant progression (7–11). Identifying key factors that influence tumor invasion and linking them to clinical stage, prognosis, and the immune microenvironment is a central challenge (12–16). Accordingly, data-driven subtyping that integrates machine learning with multi-omics analyses to identify stable and reproducible candidate molecules from high-dimensional features represents an important route toward clinical application and translation (2, 17, 18).

In this study, we used invasion-related genes to perform consensus clustering of gastric cancer and obtained molecular subtypes, followed by a systematic multi-omics evaluation of their functional differences and clinical relevance. We then combined WGCNA with multiple machine-learning models to screen key candidate genes and profiled their cellular origin, subcluster features, and potential effects on malignant cell development at the single-cell transcriptomic level. We further characterized expression patterns and prognostic associations at the pan-cancer level, and examined genomic alterations, functional features, and impacts on the immune microenvironment through multi-omics analyses.

Our study provides comprehensive evidence identifying EFNA3 as a core molecule closely associated with the invasive phenotype in gastric cancer. EFNA3 was highly enriched in specific malignant epithelial subpopulations and exhibited progressive upregulation along pseudotime, accompanied by the activation of key programs related to cell adhesion, DNA metabolism, and the cell cycle. Pan-cancer analyses further revealed that EFNA3 is consistently overexpressed across multiple tumor types and significantly associated with tumor progression and poor survival outcomes. Moreover, elevated EFNA3 expression correlated with reduced infiltration of effector immune cells and downregulation of immune checkpoint molecules, suggesting its potential role in shaping an immunosuppressive tumor microenvironment. Genomic and epigenetic data provided additional support for its regulatory potential.

To validate these findings, we examined EFNA3 expression in clinical gastric cancer tissues and cell lines, and conducted functional assays that preliminarily confirmed its role in promoting tumor cell proliferation, migration, and growth. *In vivo* tumorigenesis experiments further supported the pro-tumorigenic effect of EFNA3 in gastric cancer progression. Collectively, these results demonstrate that EFNA3 is not only a key regulatory factor within invasion-associated molecular networks, but also a promising subtype-specific biomarker and potential therapeutic target. This work provides novel insights into the malignant progression of gastric cancer and reveals potential pan-cancer commonalities that warrant further investigation.

## Materials and methods

### Data source

Transcriptomic data and corresponding clinical information for stomach adenocarcinoma (STAD) were obtained from the TCGA STAD project (https://portal.gdc.cancer.gov/), somatic mutation counts and copy number variation (CNV) data were also sourced from TCGA. Immune cell infiltration data were retrieved from the TIMER 2.0 database. Single-cell RNA-seq data for gastric cancer were obtained from GSE163558 (19). Gene dependency (CERES) and cell line expression data were derived from DepMap/CCLE. Protein–protein interaction information was obtained from STRING, pathway annotations were based on KEGG, GO, and WikiPathways. Genomic alteration data were retrieved from cBioPortal.

### Invasion-related gene set and consensus clustering

The tumor invasion gene set was obtained from a published study (20). Using the ConsensusClusterPlus package in R, STAD patients were stratified into distinct invasion-related molecular subtypes based on the expression profiles of these genes (21).

### Weighted gene co-expression network analysis

Differentially expressed genes (DEGs) between molecular subtypes were identified using the “limma” R package, with the threshold set at |log_2_ fold change (FC)| > 1 and false discovery rate (FDR) < 0.05 (22). Subsequently, weighted gene co-expression network analysis (WGCNA) was performed using the “WGCNA” R package (23). The soft-thresholding power was optimized (β = 5) to construct a scale-free network topology and identify gene modules. The correlation between modules and clinical traits, as well as gene significance, was calculated to determine key modules highly associated with the phenotype (MEturquoise) for downstream analyses.

### Identification of key genes via multiple machine learning algorithms

Before model training, samples were randomly divided into a training set and a testing set at a 7:3 ratio using a stratified sampling strategy. All models were trained on the training set and subsequently evaluated on an independent testing set. A total of eight classical machine learning models were constructed, including GLM, DT, RF, SVM, KNN, GBM, NNET, and LASSO regression models. Model training was performed using the *caret* framework in R, and a repeated 5-fold cross-validation strategy was uniformly applied to assess model stability and reduce the risk of overfitting. Model parameters were set according to the default recommendations of the *caret* framework, in which SVM employed a radial basis function kernel, the tree-related parameters of RF and GBM were automatically optimized through the cross-validation process, and the regularization parameter of the LASSO regression model was determined by cross-validation. Model performance was comprehensively evaluated using residual distributions, reverse cumulative distributions of absolute residuals, and receiver operating characteristic (ROC) curve analysis. Furthermore, feature gene importance in each model was calculated using a permutation-based approach implemented in the DALEX package.

### Single-cell RNA-seq analysis

Single-cell transcriptomic data from the GSE163558 dataset were processed using the Seurat package (v4.3). Cells were filtered based on quality control metrics, retaining those with <25% mitochondrial gene content and >3% ribosomal gene content. The filtered cells were then normalized using the LogNormalize method, followed by identification of highly variable genes and data scaling via ScaleData. Principal component analysis (PCA) was subsequently performed to reduce dimensionality. To correct for potential batch effects across samples, Harmony integration was applied using orig.ident as the batch covariate. Cell type annotation was conducted based on canonical marker genes. EFNA3 expression patterns were visualized using Nebulosa to assess its density and spatial distribution. Using normal epithelial cells, T cells, NK cells and Myeloids as a reference, inferCNV was employed to calculate copy number variation (CNV) scores across epithelial subclusters, enabling the discrimination between malignant and non-malignant populations. Pseudotime trajectory analysis was implemented using the Slingshot algorithm wrapped in the SCP package.

### Survival analysis

Univariate Cox regression analyses were performed using the ezcox R package to evaluate the prognostic impact of EFNA3 expression across four survival endpoints in all TCGA cancer types. Kaplan–Meier survival curves were generated using the survminer R package to assess the association between EFNA3 expression levels and patient prognosis across various tumor types.

### DNA methylation analysis

Pan-cancer expression and methylation data from TCGA were obtained via the Xena Browser. Spearman correlation analysis was performed to assess the association between site-specific DNA methylation levels and EFNA3 gene expression. Heatmaps visualizing the methylation–expression correlations were generated using the pheatmap R package.

### Protein–protein interaction network and pathway enrichment analysis

High-confidence EFNA3-interacting proteins were retrieved from the STRING database and used to construct a protein–protein interaction (PPI) network in Cytoscape (24, 25). Functional enrichment analysis, including GO, KEGG, and WikiPathways, was performed for EFNA3 and its interactors using the clusterProfiler R package and related tools (26, 27). To investigate the potential association between EFNA3 expression and functional dependencies, Spearman correlation coefficients were calculated between EFNA3 expression levels and genome-wide CERES dependency scores using data from the DepMap database. Genes were ranked based on correlation strength, and gene set enrichment analysis was performed on the ranked list to identify dependency-related pathways associated with EFNA3 expression.

### Clinical specimens and tissue collection

Gastric cancer tumor tissues and paired adjacent non-tumorous tissues were obtained from patients who underwent surgical resection at Qilu Second Hospital of Shandong University. Written informed consent was obtained from all patients prior to surgery. The study protocol was approved by the Ethics Committee of Qilu Second Hospital of Shandong University. Fresh tissue samples were processed immediately after resection: portions were snap-frozen in liquid nitrogen for RNA and protein extraction, while others were fixed in 4% paraformaldehyde for histological and immunological analyses.

### Cell lines and culture conditions

Human gastric cancer cell lines (MGC-803, SGC-7901, and AGS) and the normal gastric epithelial cell line (GES-1) were obtained from the Shanghai Cell Bank, Chinese Academy of Sciences. Cells were cultured in DMEM or RPMI-1640 medium supplemented with 10% fetal bovine serum (FBS) and 1% penicillin–streptomycin under standard humidified conditions (37 °C, 5% CO_2_). All cell lines were routinely tested for mycoplasma contamination prior to experimentation and confirmed to be negative.

### RNA extraction and quantitative real-time PCR

Total RNA was extracted from tissues and cultured cells using an RNA extraction kit (Feijie Biotechnology, Shanghai, China). Reverse transcription was performed using a cDNA synthesis kit (Vazyme, Nanjing, China), followed by quantitative real-time PCR using SYBR Green qPCR Master Mix (Vazyme). Gene expression levels were normalized to ATP1N1 as the internal control, and relative expression was calculated using the 2^−ΔΔCt method.

### Western blot analysis

Protein lysates were prepared using RIPA buffer supplemented with protease and phosphatase inhibitors. Protein concentrations were determined using a BCA assay. Equal amounts of protein were separated by SDS–PAGE under the following conditions: 60 V for 30 min (stacking gel) and 120 V for 55 min (resolving gel), and then transferred onto PVDF membranes. Membranes were blocked with 5% non-fat milk and incubated overnight at 4 °C with primary antibodies (e.g., anti-EFNA3 and anti-ATP1N1), followed by HRP-conjugated secondary antibodies. Protein bands were visualized using enhanced chemiluminescence (ECL) reagents and imaged with a Tanon chemiluminescence imaging system (Shanghai, China).

### Cell proliferation assays (CCK-8 and EdU)

Cell proliferation was assessed using the Cell Counting Kit-8 (CCK-8; Dojindo). Cells were seeded into 96-well plates and incubated with CCK-8 reagent for 1–2 h, and absorbance was measured at 450 nm. For EdU incorporation assays, cells were processed according to the manufacturer’s instructions, and EdU-positive cells were quantified under a fluorescence microscope.

### Migration and invasion assays

For wound-healing assays, confluent cell monolayers were scratched with a sterile pipette tip, washed to remove detached cells, and cultured in serum-free medium. Images were captured at 0 h and 24–48 h under identical magnification, and the same wound region was recorded whenever possible. Wound closure was quantified as the percentage reduction relative to the corresponding initial wound width at 0 h for each group.

For Transwell migration assays, cells suspended in serum-free medium were seeded into the upper chambers, while the lower chambers contained medium supplemented with 10% FBS as a chemoattractant. After incubation, migrated cells were fixed, stained with crystal violet, and photographed and counted under an inverted microscope (Olympus, Japan).

### Colony formation assay

Cells were seeded at low density (300–500 cells per well) in six-well plates and cultured for 10–14 days. Colonies were fixed with methanol, stained with crystal violet, and colonies containing ≥50 cells were counted to assess clonogenic capacity.

### Organoid culture

Gastric cancer cells were embedded in Matrigel and cultured in organoid medium supplemented with EGF, Noggin, and R-spondin. Organoid formation was monitored using an inverted microscope, and organoid diameter and formation efficiency were quantified to evaluate three-dimensional growth capacity.

### Apoptosis analysis

Cell apoptosis was analyzed using Annexin V-FITC/PI dual staining followed by flow cytometry. The proportions of early and late apoptotic cells were quantified and compared between experimental groups.

### *In Vivo* xenograft model

Luciferase-labeled control and EFNA3-knockdown gastric cancer cells were subcutaneously injected into the axillary region of 4–6-week-old female BALB/c nude mice (n = 3 per group). Tumor growth was monitored weekly using *in vivo* bioluminescence imaging. At the experimental endpoint, tumor volume and weight were measured. All animal experiments were approved by the Ethics Committee of Qilu Second Hospital of Shandong University and conducted in accordance with institutional and national ethical guidelines.

### Statistical analysis

All experiments were performed independently at least three times. Data are presented as mean ± standard deviation (SD). Statistical comparisons were conducted using Student’s t-test or one-way analysis of variance (ANOVA), as appropriate (15, 28). Kaplan–Meier survival analysis was performed, and p < 0.05 was considered statistically significant. Statistical analyses were carried out using GraphPad Prism or R software.

## Results

### Identification of tumor invasion–related molecular subtypes

**Figure 1:**
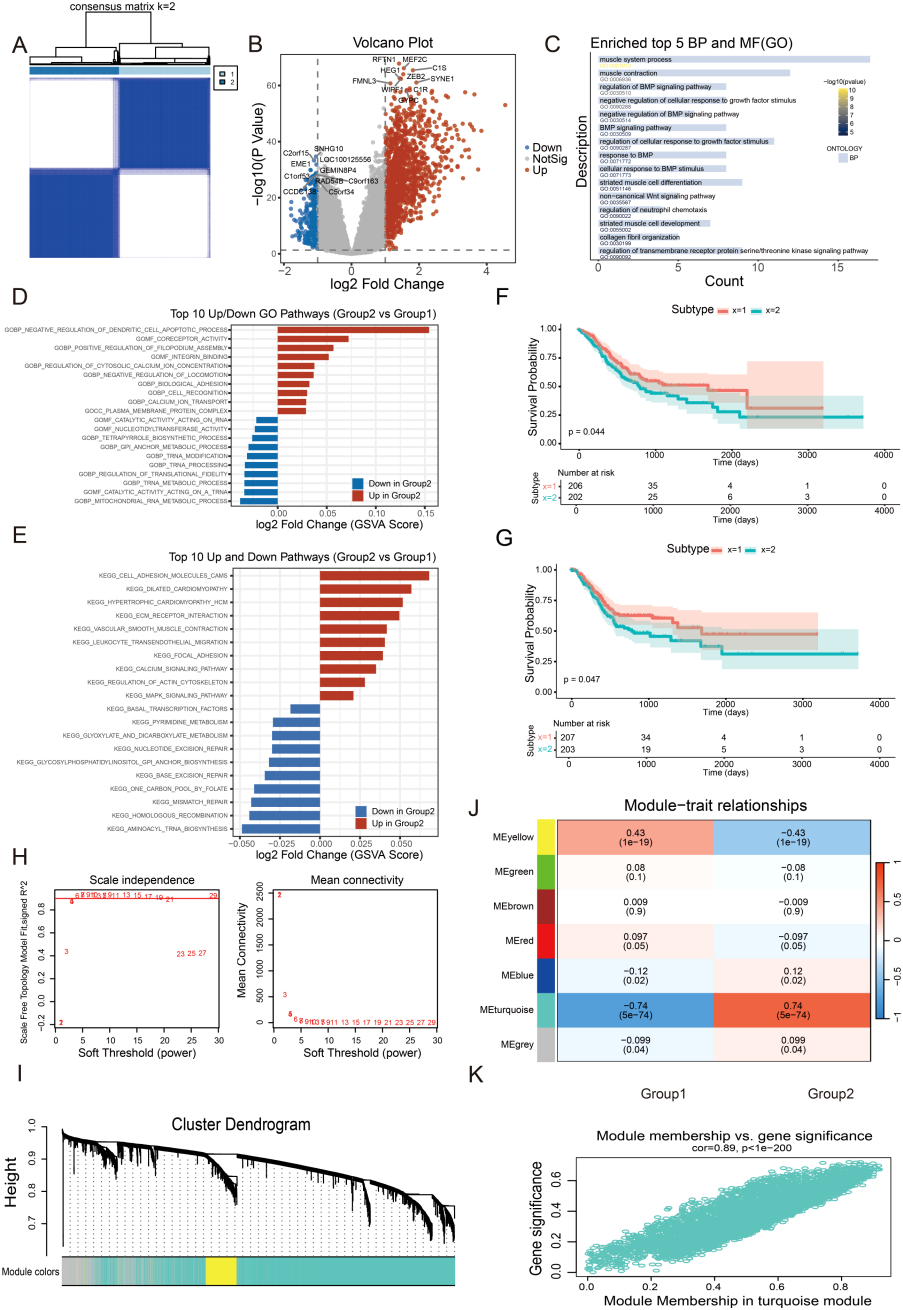
Subtype classification and functional characterization based on tumor invasion–related genes. **(A)** Consensus clustering (k = 2) classified gastric cancer samples into two molecular subtypes (C1 and C2). **(B)** Volcano plot showing differentially expressed genes between the C1 and C2 subtypes. **(C)** GO enrichment analysis of the differentially expressed genes. (**D, E**) GSVA enrichment analysis based on GO and KEGG pathways. (**F, G**) Overall survival (OS) and progression-free interval (PFI) analyses of patients in the C1 and C2 subtypes. (**H, I**) WGCNA analysis determining the soft threshold and identification of gene coexpression modules. (**J, K**) Correlation heatmap between clinical traits and coexpression modules, and the relationship between gene significance and module membership in the MEturquoise module.

Based on a curated gene set associated with tumor invasion, we performed consensus clustering analysis on gastric cancer samples. This approach stratified patients into two distinct molecular subtypes, designated as C1 and C2 (**Figure 1A**). Differential expression analysis revealed substantial transcriptional differences between the two subtypes, as illustrated by the volcano plot (**Figure 1B**). Gene Ontology (GO) enrichment analysis demonstrated that the differentially expressed genes (DEGs) were significantly enriched in several tumor-relevant biological processes, including the BMP signaling pathway, regulation of cellular response to growth factor stimulus, and the non-canonical Wnt signaling pathway (**Figure 1C**). These pathways have been extensively implicated in tumor cell proliferation, differentiation, metastasis, and maintenance of stemness in previous studies. To further dissect the functional divergence between the two subtypes, we conducted Gene Set Variation Analysis (GSVA). At the GO pathway level, the C2 subtype was characterized by upregulation of processes involved in mitochondrial RNA metabolism, tRNA processing and modification, and regulation of translational fidelity (**Figure 1D**), suggesting enhanced translational control and mitochondrial function in this subgroup. Consistent with these findings, GSVA based on the KEGG database revealed significant enrichment of pathways related to DNA damage repair and transcriptional regulation in the C2 subtype, including base/nucleotide excision repair, homologous recombination, and aminoacyl-tRNA biosynthesis (**Figure 1E**). These features collectively indicate that the C2 subtype may represent a proliferative tumor phenotype marked by elevated nucleic acid metabolism and mitochondrial activity. Survival analyses demonstrated that patients classified into the C1 subtype exhibited significantly better overall survival (OS) and progression-free interval (PFI) compared to those in the C2 subtype (**Figures 1F, G**). To identify coexpression modules highly associated with clinical phenotypes, we constructed a weighted gene coexpression network (WGCNA) using the DEGs and determined the optimal soft thresholding power to be 5 (**Figures 1H, I**). The MEturquoise module was ultimately identified and found to be significantly correlated with multiple clinical traits (**Figures 1J, K**), thereby providing a candidate basis for downstream identification of key driver genes.

### Identification of subtype-specific key genes via multiple machine learning algorithms

**Figure 2:**
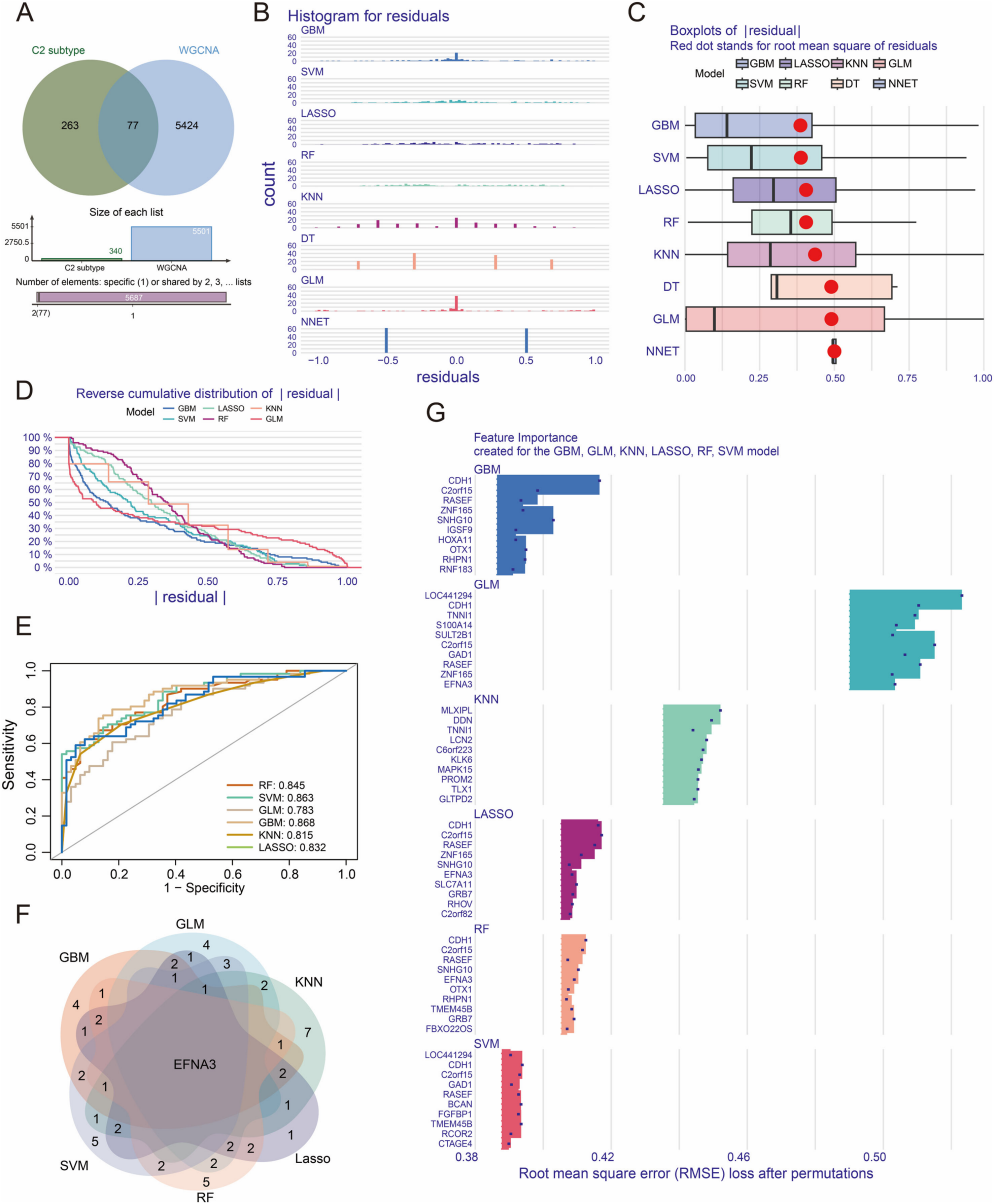
Identification of key genes in the C2 subtype using multiple machine learning algorithms. **(A)** Intersection of upregulated genes in the C2 subtype with genes from the MEturquoise module. (**B, C**) Residual distributions and root mean square error (RMSE) analyses across models. **(D)** Reverse cumulative distribution curves of absolute residuals for different models. **(E)** ROC curves and AUC comparisons among models. **(F)** Overlap of top-ranked feature genes identified by six models. **(G)** Feature importance genes derived from each model.

To further explore the key genes potentially driving the biological characteristics of the C2 subtype, we first intersected the upregulated genes in the C2 subtype with those in the MEturquoise module identified by WGCNA, yielding a core set of 77 candidate genes (**Figure 2A**). Next, leveraging the molecular subtype information from the TCGA-STAD cohort, we employed eight classical machine learning algorithms—generalized linear model (GLM), decision tree (DT), random forest (RF), support vector machine (SVM), k-nearest neighbor (KNN), gradient boosting machine (GBM), neural network (NNET), and LASSO regression, to perform feature selection and dimensionality reduction on this gene set. Analysis of residual distributions revealed that models such as GBM, SVM, RF, LASSO, KNN, and GLM exhibited more concentrated residuals and smaller fitting errors compared to the DT and NNET models, indicating superior model robustness (**Figures 2B, C**). Furthermore, reverse cumulative distribution curves of absolute residuals showed that both GBM and SVM achieved low residuals in over 90% of the samples, outperforming the other models in terms of predictive accuracy (**Figure 2D**). Receiver operating characteristic (ROC) curve analysis was conducted to evaluate classification performance across models. The area under the curve (AUC) values for GBM, SVM, RF, LASSO, KNN, and GLM all exceeded 0.75, suggesting favorable discriminative ability (**Figure 2E**). We then performed a concordance analysis of the top-ranked feature genes across models (**Figure 2G**) and extracted the top 10 genes ranked by feature importance in six of the models. Across all algorithms, EFNA3 consistently ranked among the top 20 feature-importance genes, supporting its potential as a robust and subtype-specific marker for distinguishing the C2 subgroup (**Figure 2F**).

### ScRNA-seq analysis reveals EFNA3 expression patterns in gastric cancer

**Figure 3:**
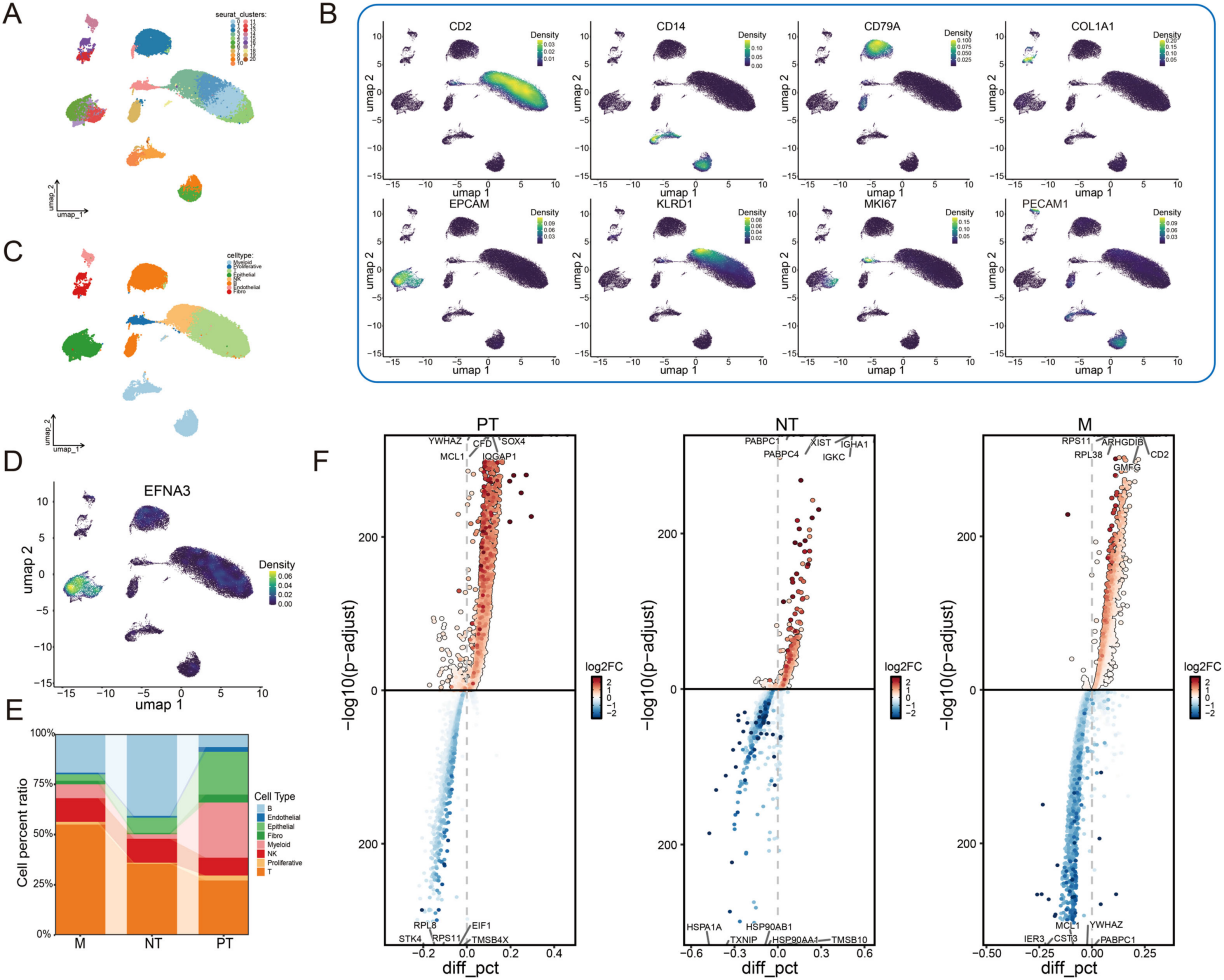
Expression pattern of EFNA3 within the tumor microenvironment of gastric cancer. **(A)** Identification of 21 cell clusters after quality control. (**B, C**) Annotation of eight major cell types based on canonical marker genes. **(D)** Expression density distribution of EFNA3 across different cell types. **(E)** Proportions of cell populations among primary, metastatic, and normal gastric tissues. **(F)** Differential gene expression analysis across tissue types.

To further elucidate the distribution of EFNA3 expression within the tumor microenvironment of gastric cancer, we performed a comprehensive analysis of the single-cell RNA sequencing dataset GSE163558. After stringent quality control, a total of 21 distinct cell clusters were identified (**Figure 3A**). Cell type annotation was carried out based on canonical marker genes, classifying all cells into eight major populations: T cells (CD2^+^), myeloid cells (CD14^+^), B cells (CD79A^+^), fibroblasts (COL1A1^+^), epithelial cells (EPCAM^+^), NK/T cells (KLRD1^+^), proliferating cells (MKI67^+^), and endothelial cells (PECAM1^+^) (**Figures 3B, C**). We then applied the Nebulosa algorithm to estimate the expression density of EFNA3 across different cell types. The results revealed that EFNA3 expression was predominantly enriched in epithelial cell populations (**Figure 3D**), suggesting its potential involvement in the development and functional regulation of tumor epithelial cells. In addition, we compared the cellular composition and transcriptional profiles among primary gastric cancer, metastatic gastric cancer, and normal gastric tissues, highlighting context-specific differences in cell proportions and gene expression (**Figures 3E, F**).

### Functional characterization of EFNA3-High malignant cells

**Figure 4:**
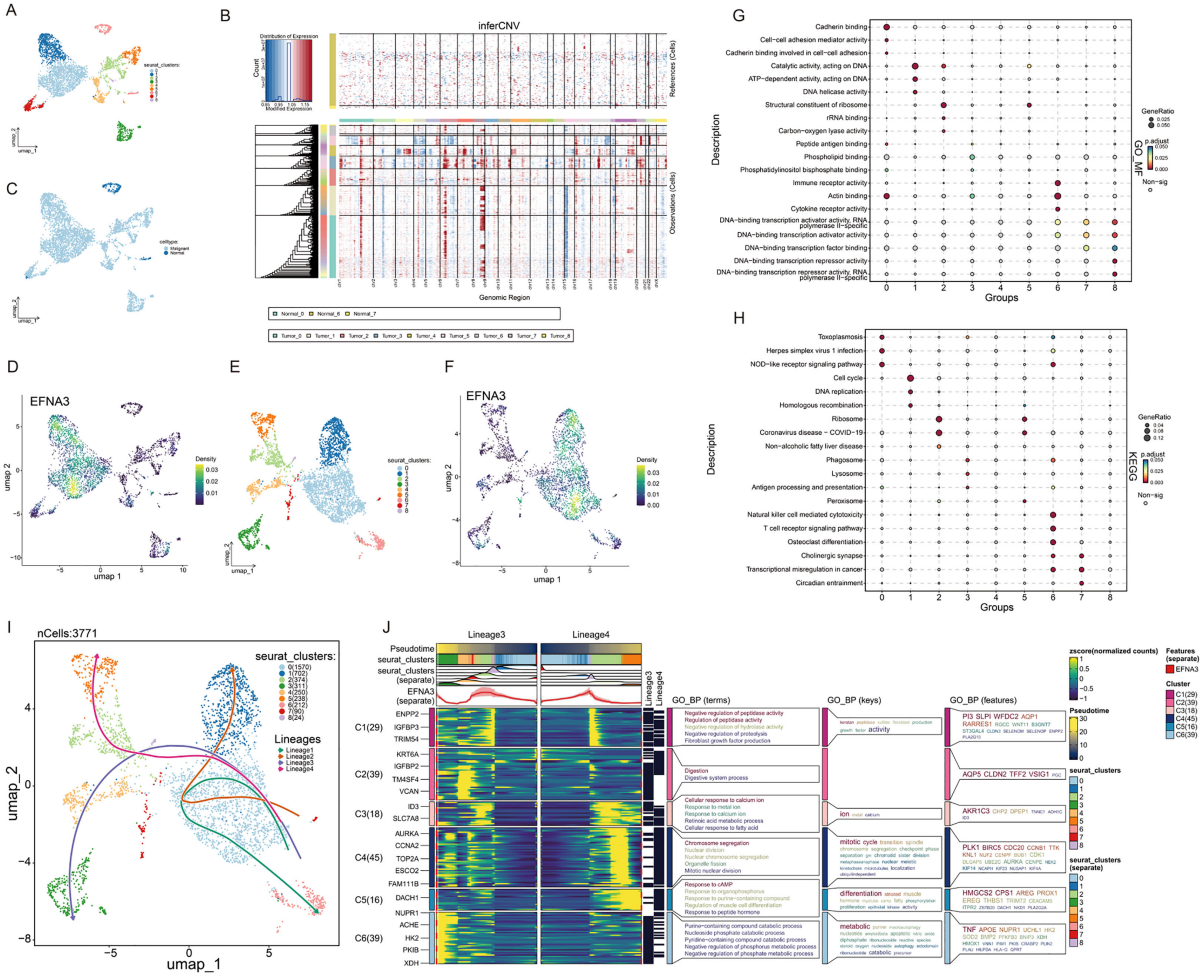
Functional characterization of EFNA3 in malignant epithelial cells. **(A)** Epithelial cells were subdivided into nine distinct clusters. **(B)** Malignant cell identification based on inferCNV analysis. **(C)** Epithelial cells were classified into malignant and normal populations. **(D)** Distribution of EFNA3 expression in epithelial cells. **(E)** Malignant epithelial cells were further clustered into nine subpopulations. **(F)** EFNA3 expression patterns across malignant epithelial clusters. **(G)** GO enrichment analysis of malignant epithelial subclusters. **(H)** KEGG pathway enrichment analysis of malignant epithelial subclusters. **(I)** Pseudotime trajectory construction of malignant epithelial cells. **(J)** Functional enrichment analysis of Lineage 3 and Lineage 4 pseudotime trajectories.

To further characterize the functional features of EFNA3 within epithelial cells, we first subclustered epithelial cells from gastric cancer samples into nine distinct subsets (**Figure 4A**). Using epithelial cells from normal tissues, T cells, NK cells and Myeloids as a reference, we applied the inferCNV algorithm to evaluate copy number variation (CNV) across epithelial subsets and calculated CNV scores for each cluster (**Figure 4B**; **Supplementary Figure 1A**). Based on CNV profiles, epithelial cells were subsequently classified into malignant cells and normal epithelial cells (**Figure 4C**). EFNA3 was found to be highly expressed in a subset of malignant epithelial cells, whereas its expression was nearly undetectable in normal epithelial populations (**Figure 4D**).

We next performed reclustering of all malignant epithelial cells and identified nine malignant subclusters (c0–c8) (**Figure 4E**). Differential expression analysis revealed distinct transcriptional signatures across these malignant subsets (**Supplementary Figure 1B**), with EFNA3 being significantly enriched in clusters c0 and c1 (**Figure 4F**). To explore the functional characteristics of these two EFNA3-high clusters, we conducted GO enrichment analysis across all malignant subsets. Clusters c0 and c1 showed significant enrichment in pathways related to cell adhesion and DNA metabolic processes, suggesting potential roles in enhanced migratory capacity and proliferative maintenance of gastric cancer cells (**Figure 4G**). KEGG pathway analysis further supported these findings, with EFNA3-high clusters exhibiting prominent activation of cell cycle and DNA replication pathways, consistent with a highly proliferative phenotype (**Figure 4H**). To further investigate the dynamic behavior of EFNA3 in malignant epithelial evolution, we constructed single-cell pseudotime trajectories and identified four major developmental lineages (**Figure 4I**; **Supplementary Figure 1C**). Compared with other lineages, EFNA3 exhibited an upward trend along the pseudotemporal trajectories of lineage 3 and lineage 4 (**Supplementary Figure 1D**), indicating its potential involvement in driving cellular transitions toward proliferative and adaptive phenotypes. Functional enrichment of terminal cells in these lineages revealed significant activation of chromosome segregation, nuclear division, DNA replication, and mitotic processes, alongside pathways related to protease inhibition, retinoic acid metabolism, and cAMP signaling (**Figure 4J**). Collectively, these findings suggest that EFNA3-high malignant subpopulations exhibit enhanced proliferative capacity, metabolic adaptability, and apoptosis resistance, underscoring EFNA3 as a potential driver of malignant progression in gastric cancer.

### Pan-cancer expression landscape and prognostic significance of EFNA3

**Figure 5:**
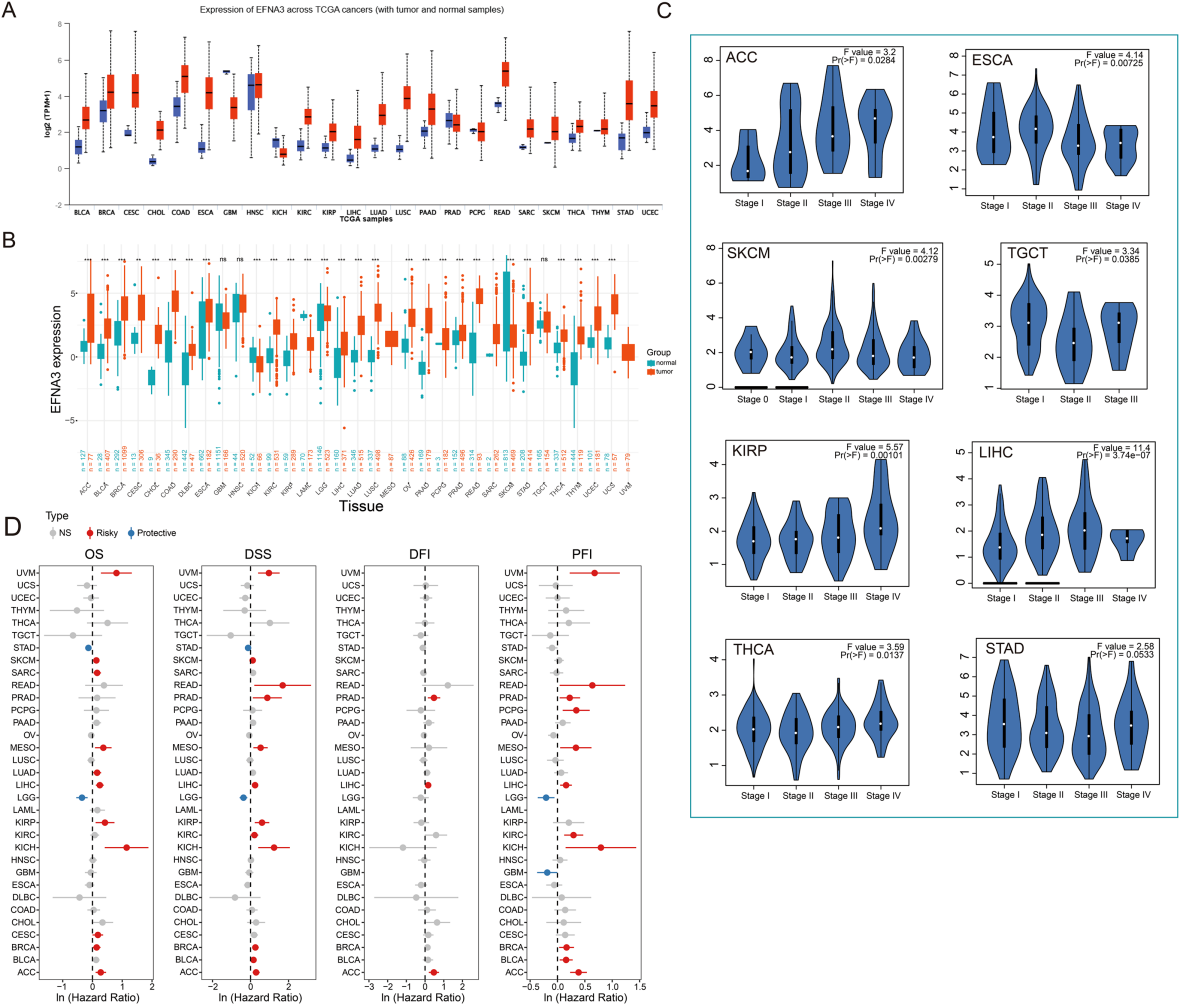
Pan-cancer expression pattern and prognostic value of EFNA3. **(A)** EFNA3 expression in tumor and normal tissues across TCGA cancers. **(B)** Differential expression of EFNA3 across various cancer types based on the integrated TCGA, GTEx, and TARGET datasets. **(C)** Variation of EFNA3 expression across clinical stages in multiple cancer types. **(D)** Cox regression analysis of EFNA3 expression and prognosis (OS, DSS, DFI, PFI) across cancers *P < 0.05, **P < 0.01, ***P < 0.001.

Given the tumor-specific expression pattern and potential pro-proliferative role of EFNA3 observed in gastric cancer, we extended our investigation to a pan-cancer context to evaluate its expression profile and clinical relevance across diverse malignancies, thereby assessing its broader applicability as a potential cancer biomarker. Preliminary analysis based on TCGA data revealed that EFNA3 is broadly upregulated in a variety of tumor tissues compared to their matched normal counterparts (**Figure 5A**). To overcome the limitation of insufficient normal samples in certain TCGA cancer types, we further integrated expression data from the TCGA, TARGET, and GTEx databases. This joint analysis confirmed that EFNA3 was significantly overexpressed in 24 cancer types, including STAD, ACC, LIHC, BLCA, and BRCA, among others (**Figure 5B**). We next examined the association between EFNA3 expression and clinical stage across cancers. Notably, EFNA3 expression showed significant stage-associated variation in ESCA, KIRP, LIHC, SKCM, TGCT, and THCA (**Figure 5C**), suggesting its possible involvement in tumor progression–related biological processes.

To assess the prognostic impact of EFNA3, we performed univariate Cox regression analyses across multiple cancer types. The results indicated that EFNA3 served as a risk factor for poor prognosis in several cancers, including UVM, SKCM, MESO, LIHC, and KIRP (**Figure 5D**). Consistently, Kaplan–Meier survival analysis revealed that high EFNA3 expression was significantly associated with worse overall survival (OS) in ACC, STAD, and UVM, as well as decreased disease-free survival (DFS) in multiple tumor types (**Supplementary Figure 2** and **S3**). Together, these findings demonstrate that EFNA3 exhibits widespread overexpression across various solid tumors and is significantly correlated with advanced clinical stage and poor patient prognosis in selected cancer types, implying a potential pan-cancer role in tumor progression. Nevertheless, further validation at the tissue level and mechanistic investigations are warranted to elucidate the functional significance and underlying regulatory mechanisms of EFNA3 in different tumor contexts.

### Putative functional mechanisms and synergistic network of EFNA3 in cancer

**Figure 6:**
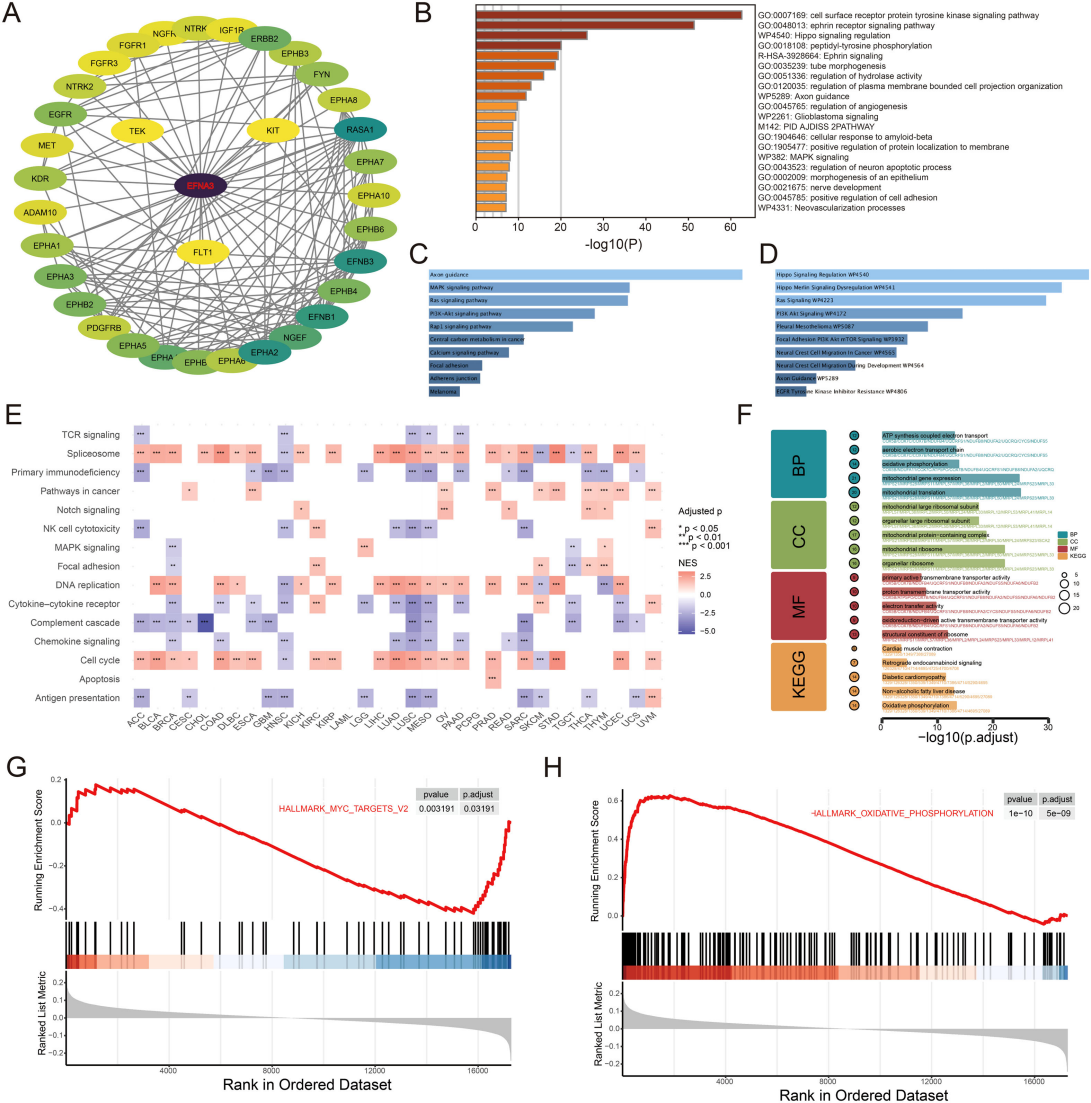
Potential Regulatory Functions of EFNA3. **(A)** PPI network of EFNA3 and its interacting proteins. **(B)** Functional enrichment analysis of EFNA3-interacting proteins. (**C, D**) KEGG and WikiPathways enrichment results of interacting proteins. **(E)** Pan-cancer GSEA enrichment analysis of EFNA3. **(F)** GO/KEGG enrichment analysis of EFNA3 dependency–negatively correlated genes. (**G, H**) GSEA enrichment analysis of EFNA3 dependency–associated genes *P < 0.05, **P < 0.01, ***P < 0.001.

To explore the potential regulatory roles of EFNA3, we first retrieved high-confidence interacting proteins from the STRING database and constructed a protein–protein interaction (PPI) network using Cytoscape (**Figure 6A**). Functional enrichment analysis of EFNA3 and its interactors revealed significant enrichment in multiple tumor-related signaling pathways, including Hippo signaling regulation, MAPK signaling, cell surface receptor protein tyrosine kinase signaling, and regulation of angiogenesis (**Figure 6B**). Further integration of KEGG and WikiPathways analysis highlighted the activation of several canonical oncogenic pathways, notably PI3K/AKT and Hippo signaling (**Figures 6C, D**). These pathways are broadly involved in cell proliferation, migration, angiogenesis, and intracellular signal transduction, suggesting that EFNA3 may promote malignant progression through orchestrating multiple cancer-associated signaling cascades. To investigate the broader functional roles of EFNA3 across cancers, we conducted gene set enrichment analysis (GSEA) at the pan-cancer level. The results showed consistent enrichment of proliferation-associated pathways, such as cell cycle and DNA replication, in nearly all cancer types (**Figure 6E**), indicating a potential role of EFNA3 in sustaining proliferative signaling during tumor development.

We further explored the dependency-related functions of EFNA3 in tumor cells by leveraging genome-wide dependency data (CERES scores) from the DepMap database. Specifically, we calculated Spearman correlation coefficients between EFNA3 expression and gene dependency scores across cancer cell lines. Functional enrichment of genes showing strong positive correlation with EFNA3 dependency revealed significant enrichment in pathways related to mitochondrial metabolism and ribosomal function (**Figure 6F**), suggesting that EFNA3 may reshape metabolic dependency by interfering with mitochondrial homeostasis and protein synthesis, thereby contributing to tumor cell survival and energy adaptation. Conversely, GSEA of negatively correlated dependency genes indicated significant enrichment in MYC target genes and oxidative phosphorylation pathways (**Figures 6G, H**). These findings suggest that EFNA3-high tumor cells may be associated with altered activity of MYC-driven proliferative programs and mitochondrial oxidative metabolism, indicating a potential context-dependent relationship and compensatory interaction involving EFNA3.

### Immunosuppressive features of EFNA3 in the pan-cancer immune microenvironment

**Figure 7:**
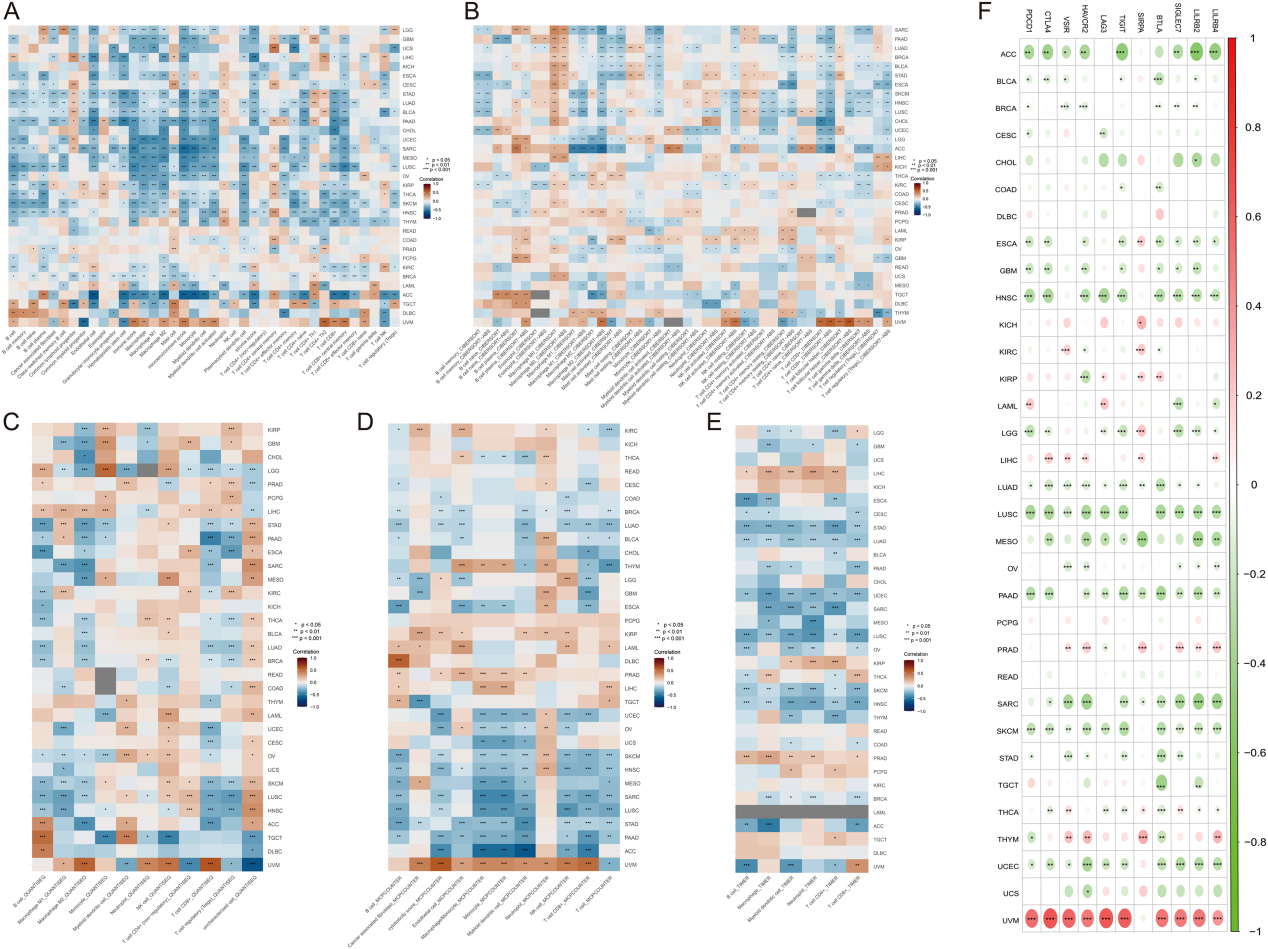
Immunosuppressive characteristics of EFNA3 in the pan-cancer tumor microenvironment. **(A–E)** Immune infiltration analyses based on XCELL, TIMER, CIBERSORT, QUANTISEQ, and MCPCOUNTER indicate that high EFNA3 expression is significantly negatively correlated with the infiltration levels of multiple immune cell types. **(F)** Correlation analysis between EFNA3 expression and immune checkpoint genes across various cancer types *P < 0.05, **P < 0.01, ***P < 0.001.

To systematically investigate the potential immunoregulatory role of EFNA3 within the tumor immune microenvironment, we performed a comprehensive correlation analysis integrating multiple immune deconvolution algorithms. Results from XCELL, TIMER, CIBERSORT, QUANTISEQ, and MCPCOUNTER consistently demonstrated that EFNA3 expression was significantly associated with reduced immune cell infiltration across multiple cancer types, with a general trend toward negative correlation. Specifically, both QUANTISEQ and XCELL analyses revealed a marked decrease in effector T-cell infiltration in tumors with high EFNA3 expression (**Figure 7A**, **7C**), a finding further validated by CIBERSORT (**Figure 7B**). Similarly, MCPCOUNTER analysis showed that high EFNA3 expression was strongly associated with diminished immune cell abundance (**Figure 7D**). Data from TIMER further confirmed these associations, showing significant negative correlations between EFNA3 expression and the infiltration levels of CD8+ T cells, CD4+ T cells, B cells, macrophages, neutrophils, and dendritic cells in several tumor types (**Figure 7E**). Collectively, these multi-method results strongly suggest that high EFNA3 expression is associated with an immunosuppressive tumor microenvironment, characterized by reduced infiltration of key immune effector cells. This implies that EFNA3 may contribute to immune evasion by attenuating tumor immunogenicity and remodeling the immune landscape.

Moreover, correlation analysis revealed that EFNA3 expression was significantly negatively associated with multiple immune checkpoint molecules, including PDCD1 (PD-1), CD274 (PD-L1), and CTLA4, in most cancer types (**Figure 7F**). These findings further indicate that EFNA3-high tumors may represent an immune “cold” phenotype, potentially associated with lower responsiveness to immune checkpoint blockade therapies.

### Pan-cancer genomic alterations of EFNA3

**Figure 8:**
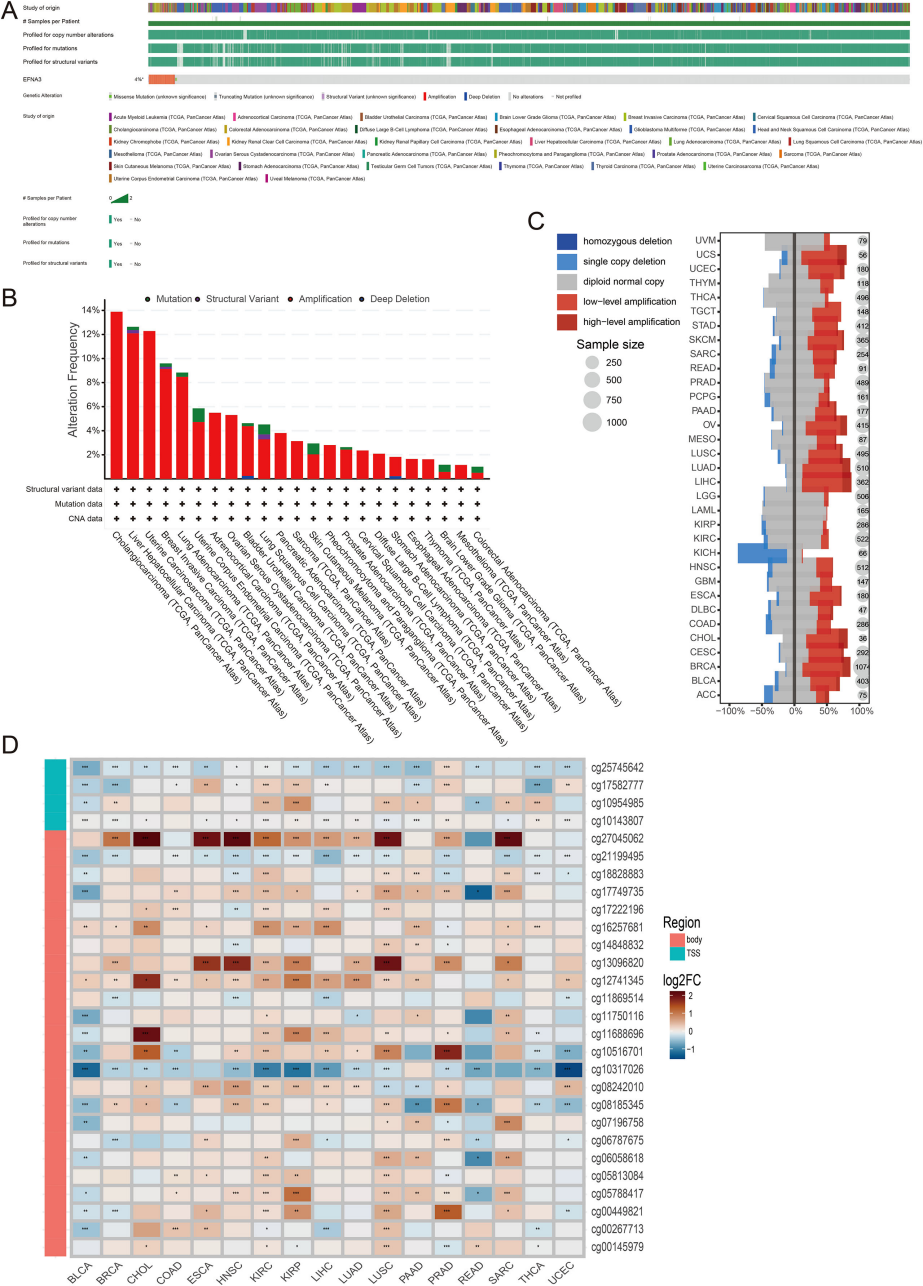
Pan-cancer genomic alterations of EFNA3. **(A)** Overview of EFNA3 genomic alterations across different cancer types. **(B)** Types and frequencies of EFNA3 genomic alterations. **(C)** Distribution of EFNA3 copy number alterations (CNA) across multiple cancers. **(D)** Correlation analysis between EFNA3 methylation sites and gene expression levels *P < 0.05, **P < 0.01, ***P < 0.001.

To investigate the genomic alteration landscape of EFNA3 across pan-cancer cohorts, we queried the cBioPortal database. The analysis revealed that EFNA3 alterations occurred in approximately 4% of tumors (**Figure 8A**). Among the different types of genomic alterations, amplification was identified as the most prevalent event in nearly all cancer types (**Figure 8B**). Specifically, low-level amplification represented the dominant subtype, whereas KICH exhibited the highest frequency of single-copy deletion (**Figure 8C**).

Promoter methylation is a critical epigenetic mechanism that regulates gene transcription. To assess its potential involvement in EFNA3 expression, we examined the correlation between EFNA3 expression levels and DNA methylation at multiple CpG sites. Notably, methylation at cg25745642 exhibited a significant negative correlation with EFNA3 expression across most cancers, suggesting that promoter hypermethylation may serve as a regulatory mechanism modulating EFNA3 transcription (**Figure 8D**).

### EFNA3 as a key regulator of gastric cancer cell growth, motility, and tumorigenicity

**Figure 9:**
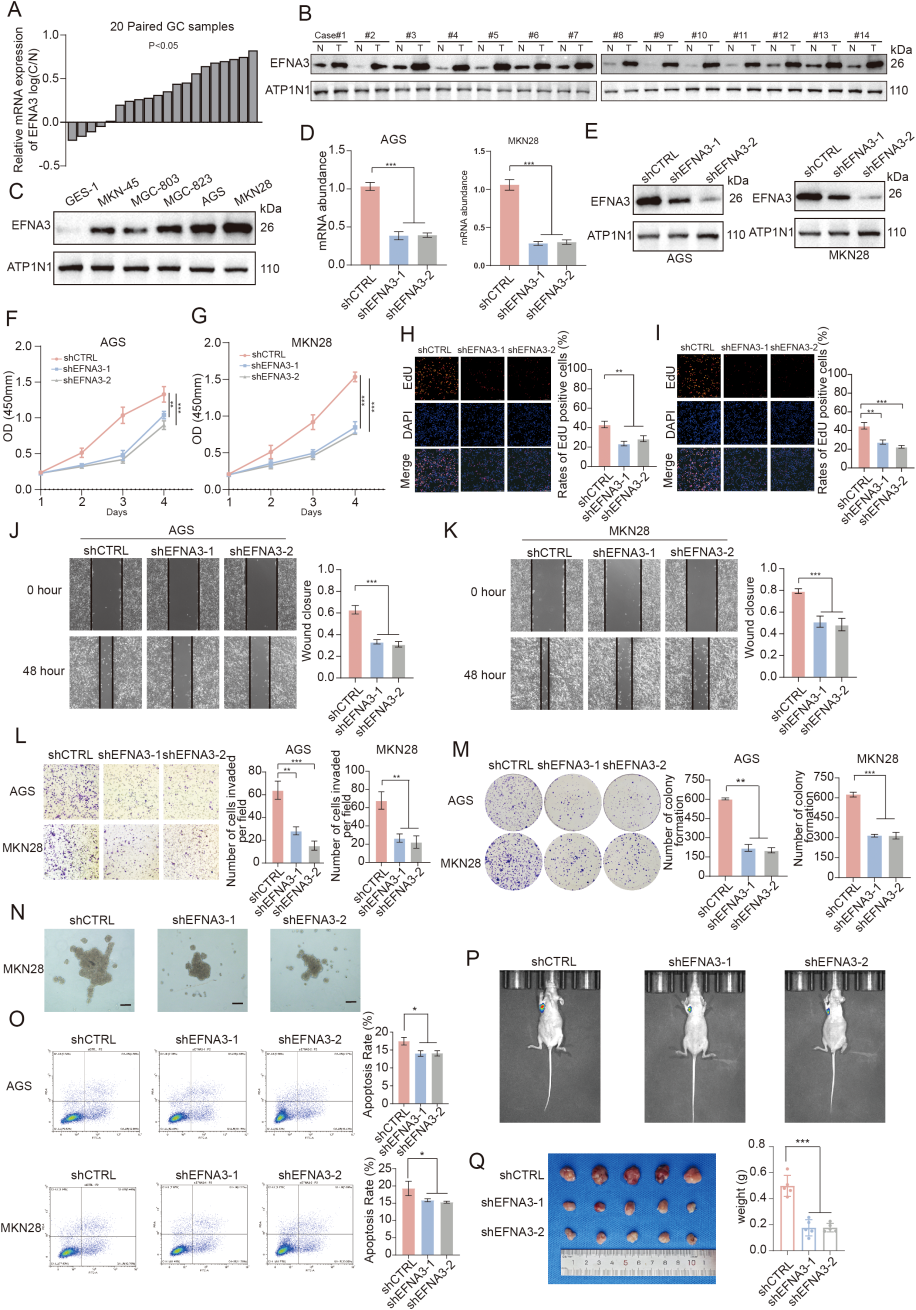
EFNA3 as a key regulator of gastric cancer cell growth, motility, and tumorigenicity. **(A)** qRT-PCR analysis of EFNA3 mRNA expression in tumor tissues and matched adjacent non-tumorous gastric tissues from gastric cancer patients (n = 20). **(B)** Western blot analysis of EFNA3 protein levels in 14 paired gastric tumor and adjacent normal tissues. ACTB served as a loading control. **(C)** EFNA3 protein expression in normal gastric epithelial cells (GES-1) and multiple gastric cancer cell lines (MGC-803, AGS, SGC-7901) as assessed by western blotting. (**D, E**) Validation of EFNA3 knockdown efficiency in gastric cancer cell lines at the mRNA **(D)** and protein **(E)** levels using qRT-PCR and western blotting, respectively. (**F, G)** CCK-8 assays demonstrating reduced proliferation in EFNA3-knockdown cells over time compared to control cells. (**H, I**) EdU incorporation assays showing decreased DNA synthesis and proliferation in EFNA3-deficient cells. EdU-positive cells were visualized and quantified. (**J, K**) Wound healing assays indicating impaired migration in EFNA3-knockdown cells. Representative images and quantified wound closure rates are shown. **(L)** Transwell migration assays confirming reduced migratory capacity following EFNA3 knockdown. Migrated cells were stained and counted. **(M)** Colony formation assays demonstrating a marked reduction in clonogenic ability in EFNA3-deficient cells after 10–14 days of culture. **(N)** Three-dimensional organoid culture showing significantly smaller average organoid diameter and reduced formation efficiency in EFNA3-knockdown cells. **(O)** Flow cytometric analysis of apoptosis using Annexin V-FITC/PI staining revealed increased apoptotic rates in EFNA3-silenced cells. **(P)***In vivo* bioluminescence imaging of xenograft tumors derived from luciferase-labeled control and EFNA3-knockdown gastric cancer cells in nude mice (axillary injection). EFNA3 knockdown tumors exhibited reduced signal intensity. **(Q)** Quantification of tumor volume and tumor weight at endpoint showing significant suppression of *in vivo* tumor growth in the EFNA3-knockdown group **P < 0.01, ***P < 0.001.

To validate our previous findings, we examined the RNA expression levels of EFNA3 in tumor tissues and matched adjacent non-tumorous tissues obtained from gastric cancer patients diagnosed at the Second Qilu Hospital of Shandong University (**Figure 9A**). The results revealed a significant upregulation of EFNA3 in the vast majority of tumor samples, suggesting its potential involvement in gastric tumorigenesis. Consistently, Western blot analysis of 14 paired gastric cancer and adjacent tissues demonstrated a markedly elevated protein expression of EFNA3 in tumor tissues (**Figure 9B**). *In vitro*, EFNA3 expression was also significantly higher in multiple gastric cancer cell lines compared to normal gastric epithelial cells, further supporting its overexpression in gastric cancer (**Figure 9C**). To investigate the functional role of EFNA3, we generated EFNA3-knockdown gastric cancer cell lines and confirmed the knockdown efficiency at both mRNA and protein levels (**Figures 9D, E**). Cell Counting Kit-8 (CCK-8) assays showed that EFNA3 knockdown significantly inhibited the proliferative capacity of gastric cancer cells (**Figures 9F, G**). Similarly, EdU incorporation assays indicated a marked reduction in proliferation rates following EFNA3 suppression (**Figures 9H, I**). Wound healing assays demonstrated impaired migratory ability in EFNA3-deficient cells (**Figures 9J, K**), and this finding was corroborated by Transwell migration assays (**Figure 9L**). Colony formation assays further confirmed that EFNA3 knockdown markedly suppressed the clonogenic potential of gastric cancer cells (**Figure 9M**).

Furthermore, three-dimensional organoid models constructed from control and EFNA3-knockdown gastric cancer cells revealed that suppression of EFNA3 led to a significant decrease in both average organoid diameter and formation efficiency, indicating a critical role in tumor growth under 3D conditions (**Figure 9N**). Apoptosis analysis showed an increase in apoptotic rates in EFNA3-deficient cells, suggesting EFNA3 may contribute to cell survival (**Figure 9O**). To evaluate the *in vivo* effects of EFNA3 on gastric cancer progression, luciferase-labeled control and EFNA3-knockdown gastric cancer cells were subcutaneously injected into nude mice. *In vivo* bioluminescence imaging demonstrated a substantial reduction in tumor signal intensity in the EFNA3-knockdown group (**Figure 9P**). Correspondingly, tumor volume and weight were significantly reduced in these mice, providing strong *in vivo* evidence that EFNA3 promotes tumor growth and progression in gastric cancer (**Figure 9Q**).

## Discussion

Gastric cancer ranks as the fifth most prevalent malignancy worldwide and represents a significant global health burden due to its high incidence and mortality rates (3, 29, 30). The pronounced molecular heterogeneity of GC, alongside its aggressive metastatic and invasive behavior, contributes substantially to poor clinical outcomes (31–34). Therefore, elucidating the molecular subtypes associated with tumor invasiveness and identifying key regulatory drivers is critical for improving prognostic stratification and advancing precision oncology in GC.

In this study, GC patients were stratified based on an invasion-associated gene set, leading to the identification of two distinct molecular subtypes. Among them, the C2 subtype was characterized by enrichment in proliferation-related pathways, including DNA repair, transcription, and translation, and was associated with significantly worse survival outcomes. WGCNA further revealed a gene module most correlated with the invasive phenotype. To identify candidate invasion drivers, multiple machine learning algorithms were applied to this module, collectively nominating EFNA3 as a putative key regulator. Previous studies have implicated EFNA3 in the regulation of cell proliferation, migration, and metabolism in cancers such as lung and bladder cancer, supporting its potential as a tumor biomarker (35–39). However, EFNA3 has not yet been systematically characterized within an invasion-associated molecular subtype framework with multi-level validation across multi-omics. Multi-omics analyses demonstrated that EFNA3 is significantly upregulated at both the transcriptomic and translational levels in GC, findings validated across multiple independent public cohorts and in-house clinical specimens. Single-cell transcriptomic profiling revealed that EFNA3 expression is primarily enriched in specific subsets of malignant epithelial cells, which exhibit enhanced proliferative and migratory/adhesive characteristics, suggesting a functional role for EFNA3 in driving tumor invasiveness. Pseudotime trajectory analysis further delineated four developmental lineages of malignant cells, with EFNA3 expression markedly upregulated along Lineage 3 and Lineage 4, both of which displayed heightened cell cycle activity—underscoring its potential involvement in malignant progression. These findings are aligned with prior reports highlighting the role of Ephrin ligands and receptors in modulating cell adhesion, migration, and angiogenesis (28, 40–44).

Pan-cancer analyses indicated that EFNA3 is broadly overexpressed across multiple tumor types and is significantly associated with unfavorable prognosis. EFNA3 expression also showed strong correlations with clinical staging in various cancers, implying its role in tumor progression. Gene set enrichment analysis (GSEA) consistently revealed that EFNA3 is associated with cell cycle and DNA replication pathways. Dependency-based enrichment analysis suggested that tumors with high EFNA3 expression exhibit reduced reliance on MYC signaling and oxidative phosphorylation, potentially indicating a shift in metabolic dependencies. Furthermore, immune infiltration analyses consistently demonstrated that EFNA3-high tumors are characterized by diminished immune cell infiltration and downregulated expression of multiple immune checkpoint molecules, indicative of an immunosuppressive tumor microenvironment. These data suggest that EFNA3 may not only drive proliferative and adhesive reprogramming but also promote immune evasion by reducing immunogenicity, highlighting its potential as a therapeutic target in immuno-oncology. Functional validation using both *in vitro* and *in vivo* models confirmed that EFNA3 knockdown significantly suppressed GC cell proliferation and migration, and attenuated tumor growth in subcutaneous xenograft models, reinforcing its critical role in tumor invasion and progression.

Nevertheless, this study has several limitations. Although both *in vitro* and *in vivo* experiments support the oncogenic role of EFNA3 in GC, its precise molecular mechanisms remain to be further elucidated, and the current evidence primarily supports its association with malignant phenotypes rather than definitively establishing EFNA3 as a direct driver of malignant progression. In addition, immune infiltration analyses suggest that EFNA3 may participate in tumor immune regulation and responses to immunotherapy. However, the reduced immune checkpoint expression and decreased immune infiltration observed in the EFNA3-high group may also indicate an immune-desert-like phenotype, which requires further clarification in larger real-world cohorts and prospective studies. Furthermore, although pan-cancer analyses revealed EFNA3-associated expression patterns across multiple malignancies, these findings are mainly based on correlative bioinformatic observations and should therefore be interpreted cautiously. EFNA3 exhibits expression heterogeneity across different cancer types, suggesting potential tumor-type specificity. Its mechanistic roles and translational relevance in distinct malignancies warrant further investigation.

Overall, this study identifies EFNA3 as a molecule associated with invasion-related heterogeneity in GC and highlights its potential relevance in tumor progression and therapeutic stratification. These findings provide a basis for future mechanistic studies and support further exploration of EFNA3 in translational oncology.

## Data Availability

The original contributions presented in the study are included in the article/**Supplementary Material**. Further inquiries can be directed to the corresponding author.

## Glossary

GC: gastric cancer
TCGA: The Cancer Genome Atlas
STAD: stomach adenocarcinoma
CNV: copy number variation
DEGs: differentially expressed genes
WGCNA: weighted gene co-expression network analysis
PCA: principal component analysis
ROC: receiver operating characteristic
AUC: area under the curve
SVM: support vector machine
KNN: k-nearest neighbors
GBM: gradient boosting machine
NNET: neural network
LASSO: least absolute shrinkage and selection operator
PPI: protein–protein interaction
qRT-PCR: quantitative real-time polymerase chain reaction
CCK-8: Cell Counting Kit-8
EdU: 5-ethynyl-2′-deoxyuridine
ECL: enhanced chemiluminescence
FBS: fetal bovine serum
EGF: epidermal growth factor
PI: propidium iodide
FITC: fluorescein isothiocyanate
IACUC: Institutional Animal Care and Use Committee.
