# Supplementary material for: Integrated multi-omics and machine learning identify EFNA3 as a key biomarker of tumor invasion
Source: Front Immunol. 2026 Apr 2;17:1742502. doi: 10.3389/fimmu.2026.1742502 (PMC13083136; doi:10.3389/fimmu.2026.1742502)
Supplement: Supplementary file 1 [file DataSheet1.pdf]

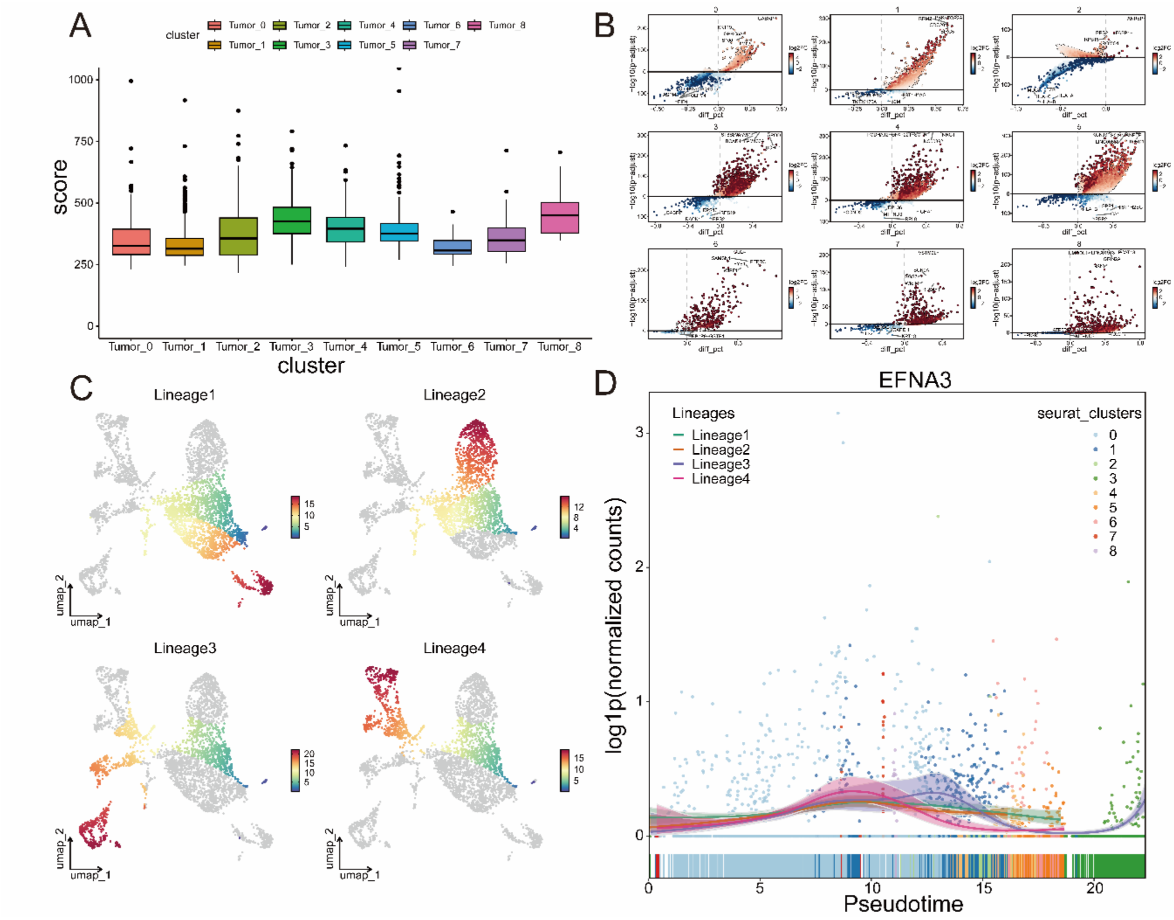

Figure S1. Functional characterization of EFNA3 in the gastric cancer tumor microenvironment. (A) CNV scores of different malignant cell subpopulations. **(B)** Differentially expressed genes among distinct malignant subpopulations. **(C)** Distinct pseudotime trajectories. **(D)** Expression dynamics of EFNA3 along different pseudotime trajectories.

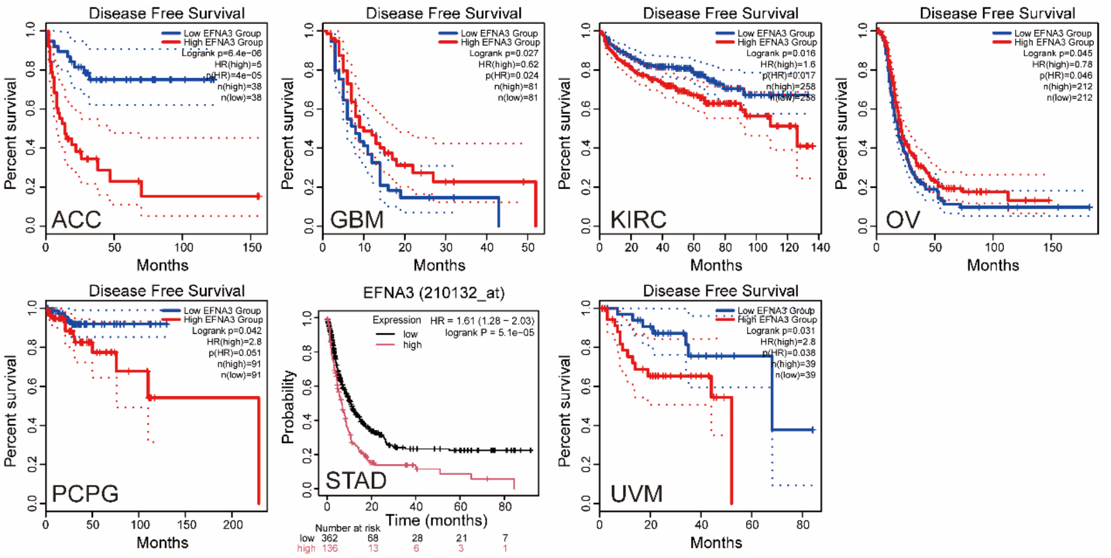

Figure S2. Impact of EFNA3 on disease-free survival (DFS) in patients with various cancers.

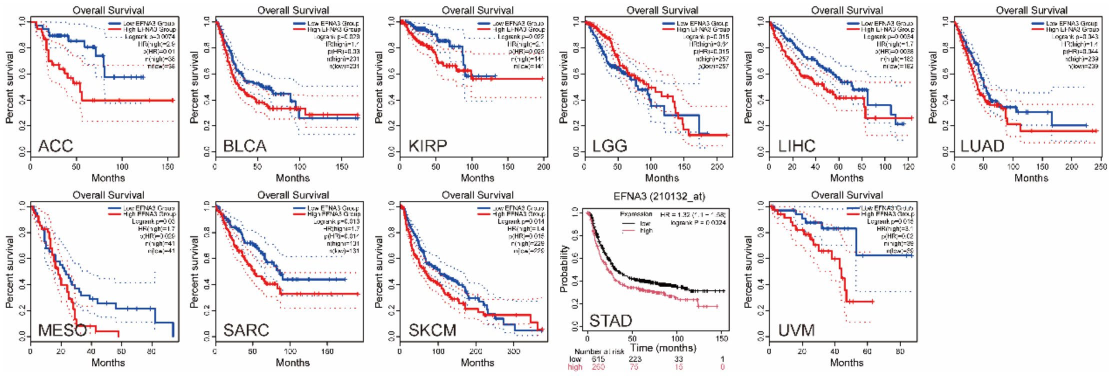

Figure S3. Impact of EFNA3 on overall survival (OS) in patients with various cancers.
